# Assessment of the Impact of Early Diagnosis and Early Treatment in the Integrated Control of East Coast Fever (ECF) Involving Acquired Immunity Induced by Natural Infection in Ankole Cattle

**DOI:** 10.3390/pathogens12010115

**Published:** 2023-01-10

**Authors:** Ann Nanteza, Zachary Nsadha, Julius Nsubuga, Stephen Oligo, Anne Kazibwe, Clare Terundaja, Enock Matovu, George William Lubega

**Affiliations:** College of Veterinary Medicine, Animal Resources and Biosecurity, Makerere University, Kampala P.O. Box 7062, Uganda

**Keywords:** *Theileria parva*, early diagnosis and treatment

## Abstract

The integrated control of East Coast fever (ECF) by early diagnosis and treatment involving acquired immunity induced by natural infection in Ankole cattle was assessed. A longitudinal study was carried out in Kiruhura district, southwestern Uganda for six months on 244 Ankole breed of cattle from 18 herds under natural tick challenge and relaxed tick control measures. Calves aged three to six months old were recruited and monitored daily by farmers for detection of ECF clinical symptoms. The reported sick animals were treated using Buparvaquone and treatment outcome determined. Monthly follow-ups and blood collections were done to monitor ECF status. Blood was analyzed for *Theileria parva* parasites by microscopy, DNA by polymerase chain reaction (PCR) and antibodies by enzyme-linked immunosorbent assay (ELISA). The overall prevalence of ECF clinical disease within six months period was 30.3% (74). The major symptoms of early clinical ECF disease were fever and enlarged parotid or prescapular lymph nodes. Clinical cases were categorized as mild, 24% (18) or moderate, 76% (56). There was an overall recovery rate of 100% (74) of the ECF cases whereby 94.6% (70) recovered promptly and 5.4% (4) recovered slowly. Based on blood analysis, prevalence of ECF at baseline was 3.7% (9) by microscopy, 31.1% (76) by PCR and 38.1% (93) by ELISA. A significant increase (*p* < 0.05) was shown by the increased number of calves with *T. parva* specific antibodies in the sera from 38.1% at baseline to 68.8% after six months. High antibody levels (positive percentage ≥ 50%) were detected in all ECF-treated and recovered calves at the end of six months. The acquired immunity to ECF was high in treated and recovered cattle, indicating that natural exposure to infection, accurate early diagnosis and effective treatment enhance development of immune-protection in indigenous cattle in an endemic area. The prominent early clinical symptoms for ECF could be exploited in the development of decision support tools for chemotherapy and other integrated control measures.

## 1. Introduction

Tick-borne diseases such as theilerioses that are endemic in large parts of the tropical and sub-tropical regions of the developing world are ranked high in terms of their impact on the livelihood of resource poor cattle farming communities. East Coast fever (ECF), which is caused by the intracellular protozoan parasite, *T. parva*, is a devastating tick-borne disease of cattle in eastern, central and southern Africa. The parasite induces a lymphoproliferative syndrome, resulting in rapid deaths in susceptible animals. The disease was estimated to be responsible for US$170 million of economic loss annually [1], and approximately one million cattle die of ECF each year. It is particularly severe in exotic (*Bos taurus*) cattle, but it also causes high mortality in young Ankole or Zebu (*Bos indicus*) cattle in pastoralist or extensive farming systems. Currently, ECF is controlled primarily through limiting *T. parva* infection with the tick vector, *Rhipicephalus appendiculatus*, (*R*. *appendiculatus*) using chemical acaricides. However, this method has major drawbacks, including the development of resistance by ticks, food-safety concerns and environmental contamination resulting from toxic residues [2]. 

After nearly a century of acaricide utilization, it is widely believed that acaricides alone do not provide a sustainable solution to tick- and tick-borne disease (T&TBDs) control [3]. Chemical acaricides, treatment of infected cattle using therapeutic drugs (*Buparvaquone* and *Parvaquone*), grazing management (burning of tick breeding sites and zero-grazing), and cross-breeding are the primary methods of ECF control [4,5,6]. However, these methods are neither very effective nor sustainable. The consistent use of acaricides is generally too costly for smallholder cattle keepers [4] and is unsustainable because of the increasing drug resistance in the targeted ticks [7]. Treating sick animals using therapeutic drugs is often ineffective, as the method has to be done in the early stages of infection [8]. In most cases, calves that recover from the disease remain stunted, and are carriers of ECF parasites [3]. Besides, ECF treatment drugs are also generally too costly for smallholder farmers [9]. 

Apparently, a highly effective ‘infection and treatment method’(ITM) live vaccine, “Muguga Cocktail”, based on the injection of a potentially lethal dose of sporozoites together with a long-acting dose of tetracycline has long been commercially available for control of ECF. Despite its efficacy, the ITM has historically been used only on a limited scale because of logistical and policy constraints. This vaccine, therefore, has not been used in some countries where the disease is endemic. In Uganda, the vaccine is not officially registered by National Drug Authority (NDA) although, two veterinary drug pharmacies have been licensed to import and supply the vaccine and it is used irregularly and at a very low rate (less than 5%) by private commercial cattle farmers with exotic dairy cattle that are very productive but very susceptible to ECF. The vaccine is also not cost-effective for farmers with indigenous cattle breeds (approximately USD 15/animal/dose). Additionally, the vaccine was developed several decades ago and produced by costly methods that could not attract manufacturers to target the low resource markets. 

It is known that effective and rational treatment of ECF requires early and accurate diagnosis. Apart from acaricide application, the second line of ECF control is diagnosis and treatment of infected animals. For treatment to be effective, diagnosis must be done early when parasites are still few and this requires sensitive and specific diagnostic techniques. However, diagnosis of ECF in the field to date depends mainly on the detection of parasites in Giemsa-stained blood smears, which may not be a true indicator of clinical ECF. This is particularly common in ECF-endemic areas in which the detection of parasites not only indicates an active infection but may also indicate either a slow proliferation of macro-schizont-infected lymphocyte or a carrier state of the animal [10]. Thus, diagnosis of ECF cases in such areas may be confusing, unrealistic, and even not practical. Animals which recover from ECF have a long-lasting protection against virulent homologous challenge and the level of protection is not related to the severity of the initial reaction. In an ECF endemic area with indigenous cattle breeds, the maternally inherited antibodies decay at around three months of age. Thus, weaning at three to four months exposes calves to infection when they co-graze with the dams, when they are highly susceptible to ECF. This results in high mortality which decreases when the antibody levels increase after exposure to infection. This study assessed impact of early diagnosis and early treatment in the integrated control of East Coast fever (ECF) involving acquired immunity induced by natural infection in Ankole cattle. 

## 2. Materials and Methods

### 2.1. Study Sites and Animals

**Figure 1:**
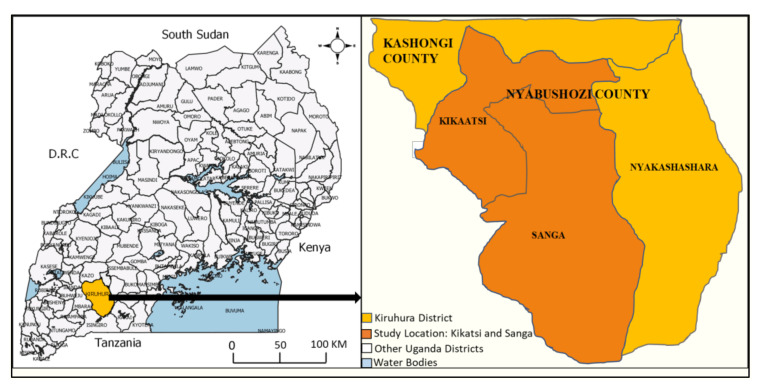
Location Map of the Study Areas. To the left is the map of Uganda with location of Kiruhura district and to the right is an extract of the map of Kiruhura district indicating Sanga and Kikatsi sub-counties study locations. The maps were obtained courtesy of Uganda Bureau of Statistics and then modified.

The study was conducted in two sub-counties (Sanga and Kikatsi) in Kiruhura District in south-western Uganda (Figure 1). Eighteen (18) cattle farms were purposively selected based on the extensive animal management practices with or without paddocks and history of occurrence of T&TBDs. Nine (9) farms were selected from each of the two study sub-counties. Each of the selected farms had a minimum of 100 cattle to be able to select at least 13 calves for the study. The study period included a wet and dry season (August, 2020 to January, 2021). Animals used were the Ankole breed of cattle (3–6 months old; males and females). At the beginning of the study, 244 calves were recruited and ear-tagged for subsequent identification purposes. The calves from each study farm were randomly selected with the consent of the animal owners. A large number of animals (244) were recruited into the study to determine ECF prevalence, efficiency of early diagnosis and early treatment of ECF and also cater for animals that could not be followed up for consecutive six months due to non-compliance by farmers, animal deaths or loss. 

### 2.2. Study Design 

Prior to the commencement of the study, district local council (LC) leaders including the District Veterinary Officer (DVO) and farmers with Ankole cattle that are kept under extensive management systems were informed about the study to assess the ECF early diagnosis and early treatment performance and its benefits. It was a longitudinal and case study design whereby the Ankole breed of cattle calves (3–6 months old) that were recruited in the study were monitored daily by the farmers for clinical ECF case detection and further assessed for treatment outcome. Questionnaires were administered to assess farmer and farm characteristics in relation to farm management and ECF control by chemotherapy, tick control using acaricides and/or other approaches. Farmers were trained by the study team members and assigned to monitor the study calves for clinical symptoms of ECF throughout the study period. The calves that were tentatively diagnosed as ECF-positive based on clinical symptoms were reported to the field veterinary officers who confirmed that they are ECF clinical cases and immediately treated them using the curative drug, Buparvaquone (Butalex^R^). The treated ECF sick animals were further monitored by the farmers and field veterinary officers over 10 days to evaluate the treatment outcome. Recovered cattle were also assessed further for 14 days post-recovery for incidence of re-occurrence of ECF disease. 

Cattle were monitored and sampled at monthly intervals by the study team members to determine the ECF clinical status, parasitemia, antibodies and parasite DNA. Three to four milliliters (3–4 mL) of blood was collected from the jugular vein of each animal into EDTA vacutainer tubes for genomic DNA extraction, thin blood smear preparation and packed cell volume (PCV) determination. Similarly, blood was also collected in plain vacutainer tubes for sera recovery for the subsequent *T. parva* antibody detection by ELISA [11,12]. The blood was screened parasitologically by microscopy and molecularly by PCR for *T. parva* infections once a month for six consecutive months. Additionally, serological analysis for *T. parva* antibody detection was assessed twice during the study (baseline and end of six months). 

### 2.3. Clinical Diagnosis and Treatment of East Coast Fever Cases

A calf was suspected to be an ECF clinical case based on the following symptoms: enlargement of superficial lymph nodes (particularly the parotid and/or pre-scapular), high body (rectal) temperature/fever (≥39.5 °C), dullness and respiratory distress [13]. Participating farmers were trained to identify and record these symptoms as soon as they were observed and to immediately inform the field veterinary officers. Animals that were confirmed to have ECF by the veterinary officer were treated using Buparvaquone (Butalexâ, Pitman-Moore). The drug was administered intramuscularly in the neck region at a dose rate of 2.5 mg/kg body weight (as per manufacturer’s recommendations) and repeated at the same dosage after 48 h. Treated animals were monitored for 24 days to assess treatment outcome or re-occurrence of disease. The severity of the disease at the time of treatment was categorized based on the criteria set for ECF clinical reactions (mild, moderate or severe) [13]. Responses to treatment were recorded according to Matovelo et al. [13] as prompt/rapid, slow/sluggish, or dead. A “prompt” response was recorded when animal showed marked clinical improvement by reduction of rectal temperature by 1 °C within 48 h after the first dose of treatment. A “slow” or “sluggish” response was recorded when treated animals did not show a marked clinical improvement (decrease in body temperature of <1 °C) within 48 h post-initial treatment. The category “dead” could have been used if the animal died within the 24 days follow-up period with detection of *T. parva* parasites in either lymphocytes or erythrocytes and DNA by PCR plus the lesions suggestive of ECF at post-mortem.

### 2.4. Hematocrit/Packed Cell Volume (PCV) Analysis

The hematocrit or PCV determines the percentage of red blood cells (RBCs) in whole blood. Hematocrit determinations were manually performed by the microhematocrit method. The capillary tube was filled to the level of two-thirds to three-quarters full with well-mixed, EDTA venous blood. One end of the tube was sealed with plasticine. The filled tube was placed in the microhematocrit centrifuge, with the plugged end away from the center of the centrifuge. Centrifugation was done at a preset speed of 10,000 revolutions per minute (rpm) for 5 min. The tube was placed in the manual microhematocrit reader and read by following the manufacturer’s instructions on the microhematocrit reading device. When hematocrit determinations were below normal (˂24%), medical conditions such as anemia and leukemia may be present. Above-normal (˃48% in cattle) hematocrit determinations indicate medical conditions like dehydration. 

### 2.5. Parasitological Analysis by Microscopy

Thin blood smears were prepared using a thin drop of well mixed EDTA blood and left to air dry at room temperature. The smears were fixed in absolute methanol for 5 min, air-dried, placed in 10% Giemsa’s stain for 30 min, the stain washed off using tap water and left to air-dry. In total, 200 fields of each smear were examined at 1000× magnification using a light microscope. Smears were assessed for piroplasm parasitemia. They were scored as either negative or positive and expressed in percentages for expression of disease prevalence. 

### 2.6. Blood Sample Storage and Preparation for Molecular Analysis 

Whole blood samples for molecular analysis were transported on ice in a cool box from the field and stored at −20 °C in the Molecular Biology Laboratory (MOBILA), COVAB, Makerere University to ensure DNA preservation. They were subsequently used for DNA extraction and molecular analysis in the same laboratory. 

### 2.7. Genomic DNA Extraction from Whole Blood 

Blood was processed for DNA extraction as described by Conrad et al., [14]. Briefly, 200 µL of thawed blood was washed three times in 0.5 mL phosphate buffered saline (PBS) (pH 7.4) in a 1.5 mL Eppendorf tube by centrifugation at 16000 g for 5 min. The cell pellet was re-suspended in 100 µL of lysis mixture (10 mM Tris-HCl, pH 8.0), 50 mM KCl, 0.5% Tween 20, 100 µg/mL of proteinase K) followed by overnight incubation at 56 °C. The mixture was then boiled for 10 min to inactivate proteinase K and centrifuged for 5 min. The supernatant (50 µL) containing DNA was aliquoted into clean tube and stored at −20 °C until use. 

### 2.8. The p104 Gene-Based PCR Screening of T. parva-Infections

The extracted genomic DNA was used for *T. parva* parasite detection. Nested PCR for an invariable region of the p104 gene (nPCRp104) was used to screen blood samples for presence of *T. parva* DNA as described by Odongo et al. [15]. The method generates a 496 bp fragment using forward and reverse primers IL3231 (5′-ATTTAAGGAACCTGACGTGACTGC-3′) and IL755 (5′-TAAGATGCCGACTATTAATGACACC-3′), respectively [16], in the first reaction. The secondary PCR uses forward and reverse primers 5′-GGCCAAGGTCTCCTTCAGATTACG-3′ and 5′-gTGGGTGTGTTTCCTCGTCATCTGC-3′, respectively to amplify a 277 bp internal fragment located between bases 2784 and 3061 of the p104 gene. Five microliters (5 µL) of genomic DNA template was used in the primary PCR while 1 µL of template (product of the primary PCR) was used for the secondary PCR. The amplification was carried out in a 25 µL reaction volume containing 1× DreamTaq Green PCR master mix (Thermo Scientific), 50 ng of each of the specific primers (Eurofins). The cycling conditions were initial denaturation at 95 °C for 5 min, followed by 35 cycles of denaturation at 95 ˚C for 1 min, annealing at 58 °C for 1 min and extension at 72 °C for 1 min then a final extension at 72 °C for 7 min before cooling at 4 °C. For the secondary PCR, the mixture components and cycling conditions were similar as the primary PCR except the annealing temperature was 55 °C for 1 min and 30 cycles were performed. Five microliters of the PCR products were separated on 2% agarose gels in Tris-acetate–EDTA (TAE) buffer containing 0.5 µg/mL ethidium bromide. The DNA bands were visualized in an UV light box and photographed. The PCR thermocycler used throughout the study was the GenAmp^®^ while the BIORAD power pac^TM^ was used for agarose gel electrophoresis. Double distilled water was used as a negative control whereas *T. parva* (Muguga strain from International Livestock Research Institute (ILRI), Kenya) was used as positive control. 

### 2.9. Serological Analysis by ELISA

Detection of serum antibodies to *T. parva* was carried out using ELISA as described by Katende et al. [12]. Antibody levels to *T. parva* were determined at two time points at baseline and end of the study. Serum samples were assayed for antibodies to *T. parva* by ELISA using recombinant polymorphic immunodominant molecule (PIM) as the antigen. The test was done at ILRI, Kenya. The Optical density (OD) readings for test sera were expressed as percentage positivity (PP) and calculated by OD (test serum)/OD (positive (+ve) control serum) × 100 and PP ≥ 20% was considered as positive for *T. parva* antibodies. 

### 2.10. Data Analysis

Data included farmer and farm characteristics obtained by questionnaire survey, Farmers’ and field veterinary officers’ records indicated ECF symptoms (swollen superficial lymph nodes, high rectal temperature/fever, dullness and respiratory distress/labored breathing) and treatment outcomes. Laboratory records indicated presence or absence of *T. parva* infections by PCR and antibody titers were determined by ELISA. Data were analyzed by STATISTIX 7 computer software using descriptive statistics. The percentages of ECF occurrence that were detected by each method were analyzed. Confirmation of positive clinical ECF cases was based on parasitological and molecular analyses by microscopy and PCR, respectively. The recovery rates of treated animals were determined. Efficacy of the study strategy for ECF control was determined and its relevance as an alternative and farmer-friendly method of immunizing cattle was assessed. 

## 3. Results

This section has been divided by subheadings. It provides a concise and precise description of the experimental results, their interpretation, as well as the experimental conclusions that can be drawn. 

### 3.1. Farmer and Farm Characteristics 

This study involved a total of 18 households/farms from two sub counties of Sanga (9) and Kikatsi (9) in Kiruhura district. The percentage of households with cattle, goats, sheep and poultry were 100%, 50%, 17% and 39%, respectively. The farmers’ socio-demographic data and farm characteristics are shown in Table 1. 

### 3.2. Symptoms and Responses to East Coast Fever Treatment

The number of calves that were diagnosed as ECF clinical cases based on farmer-recognizable symptoms within six months study period was 74 (30.3%). The presentations of symptoms varied from one animal to another, ranging from mild to moderate reactions (Table 2). Febrile reaction (high rectal temperature) and lymph node enlargement were the prominent clinical signs in early ECF cases. The other known signs of dullness and/or respiratory distress were not reported by the farmers; however, dullness was observed by the field Veterinary Officers in a few animals (seven) prior to treatment. None of the observed and reported clinical cases showed severe signs. The treatment outcomes were based on fever/rectal temperature assessment. There was an overall recovery rate of 100% (74), whereby 70 (94.6%) showed rapid or prompt response to treatment and four (5.4%) showed slow/sluggish recovery. Of all animals, 18 (100%) with mild clinical symptoms recovered promptly and the majority 52 (92.9%) of moderate clinical disease category also showed prompt recovery (Table 2). 

### 3.3. Hematocrit (Packed Cell Volume) Analysis

Packed cell volume (PCV) readings below 24% are considered as indicators of anemia in cattle. All the PCV values obtained during the six months monitoring study period were in the 28–40% range which is within the normal range (24–46%) for cattle. 

### 3.4. Prevalence of East Coast Fever Based on Laboratory Analyses

Thin blood smears from a total of 244 cattle at baseline that were examined for the presence of *T. parva* parasites using a light microscope identified nine (3.7%) animals as positive for ECF infection (Table 3) All positive results were obtained in blood samples from ECF symptomatic calves. However, during the six months follow-up period, more calves (94) showed parasites in blood and these included both ECF symptomatic (74) and asymptomatic (20). Out of the 94 cumulative (6 months period) microscopically detected cases, 51 (54.3%) were *T. parva* mono-infections, while 43 (45.7%) samples were *T. parva* and *Anaplasma marginale* mixed infections.

At baseline, nPCR analysis for *T. parva* p104 gene revealed 76 (31.1%) positive samples for parasite DNA (Table 3). All the samples that were positive by microscopy were also positive by PCR. The latter was approximately 10 times more sensitive than microscopy. More *T. parva* positive samples, 157 (64.3%) were detected by nPCR at the end of the six-month study period. 

The ELISA findings at baseline revealed 93 (38.1%) positive sera for *T. parva* antibodies (Table 3). All the positive samples by either microscopy or PCR were also positive by ELISA with some samples that were parasite negative by microscopy and PCR being positive by ELISA. The prevalence of ECF by PCR and ELISA were in the same range but significantly higher than microscopy data. At the end of the six months follow up period, all the treated and recovered cattle 74 (100%) showed high levels of antibodies (PP > 50%). At end of study, the sero-prevalence was approximately double (68.8%), which was significantly higher (*p* < 0.05) than that at baseline (38.1%) (Table 3). 

### 3.5. East Coast Fever Re-Occurrence and Mortality 

Calves (74) that recovered from ECF showed resistance to subsequent field disease challenge as none of ECF-treated cattle had a second disease episode at different time points and no fatal cases were recorded during the 6-month follow-up study period. 

## 4. Discussion

The effectiveness of early treatment of natural ECF cases as a method of protecting cattle against the disease was determined by exploring the efficiency of early detection by farmer-recognizable symptoms and immediate treatment. The integrated control of ECF by early diagnosis and treatment involving acquired immunity induced by natural infection in Ankole cattle showed good ECF control management. All the diagnosed ECF cases recovered after treatment with the anti-theilerial drug Buparvaquine, which indicated that in endemic areas, the diagnosis of ECF based on symptoms can be a reliable diagnostic marker. Appropriate recovery from clinical disease leads to ECF carrier status which could be contributing to attaining and improving endemic stability in endemic areas in indigenous cattle breeds that are less susceptible to ECF. Early diagnosis and early treatment resulted into direct protection among the treated calves. Consequently, implementation of early diagnosis and early treatment of ECF is essential to keep the disease under control. However, the curative drug (Buparvaquine) is very expensive, which makes farmers opt for cheap drugs such as Oxytetracycline, which are less effective. 

All the cattle 74 (100%) that were symptomatically diagnosed as ECF cases recovered after treatment with the anti-theilerial drug, Buparvaquine. Results from ECF treatment outcome analysis revealed that chemotherapy using early ECF symptomatic detection by farmers prevented deaths due to ECF. Early treatment leads to recovery from ECF but the animal attains a carrier status which elicits the immune system to produce antibodies that are protective. The recovery of all the early detected ECF cases suggests that in endemic areas, the diagnosis of ECF based on clinical symptoms can be a reliable diagnostic tool. A key feature of infections with Theileria parasites on the other hand is their ability to establish persistent infections in the face of immune responses that control the infection. In the case of *T. parva* and *T. annulata*, persistent infections (referred to as the carrier state) are usually not detectable microscopically but can be revealed by PCR assays and can be transmitted by ticks [16]. 

Anemia, which is best measured by PCV, is one of the key-indicators of poor health status of an individual. The PCV values within the normal range (24–48%) detected in ECF infected cases in this study can be explained by the fact that ECF does not cause anemia in its pathogenesis and furthermore, it can be explained by the abundant pasture land for the available herds at the time of the study which provided good nutrition to boost the animal’s immune system. 

Blood samples were analyzed for comparative ECF diagnosis using microscopy, PCR and ELISA. Microscopy was used because it is the standard practical method for ECF diagnosis but it has the disadvantages of poor sensitivity and specificity, especially during low parasitemia. However, it is often difficult to distinguish between parasite species. This study also revealed that blood smear examination is not useful in the diagnosis of ECF at early stages and should not be used as a sole means of defining ECF case in the absence of clinical data. Failure to detect parasites in some ECF cases, which had symptoms, may be viewed as a source of error in diagnosing ECF, especially in early stages of the disease, when parasites are still within the lymph nodes and/or only a few red blood cells (RBCs) are infected. In this respect, use of alternative methods with higher sensitivity, like PCR, may improve the detection of such parasites, especially when screening a high number of samples [17]. However, the progressive increase of piroplasm parasitemia at the end of the six months study follow-up period indicated the role of blood smears in defining carrier status of animals, which is particularly common in endemically stable areas where most recovered animals become carriers of parasites and reveal parasites in blood even without clinical disease [3,18]. 

The PCR was able to detect *T. parva* DNA in all the 74 (100%) symptomatic ECF cases. This molecular method is recognized as the most sensitive and specific method of all diagnostic tests for ECF that are currently available [15]. However, nPCR has the disadvantage of being time-consuming and having many steps in the diagnostic procedure. Furthermore, it is expensive because of complex equipment needed to run the PCR assay. These disadvantages prevent the implementation of nPCR as an alternative diagnostic test in poor countries and remote areas where ECF is mainly distributed. Thus, the use of alternative methods with higher sensitivity, like Loop Mediated Isothermal Amplification (LAMP), may improve the detection of parasites, especially when screening a large number of samples [15,18]. 

The data for the antibody response were presented as percentage positivity (PP) whereby values ≥ 20% are considered as positive for ECF. The antibody response, however, is merely an indication that the animal has recognized the presence of *T. parva* antigens either during the immunization or from field tick/parasite challenge. There was a significant increase (*p* < 0.05) in numbers of animals that were positive by ELISA from 38.1% at baseline to 68.8% after 6 months of follow-up period and the antibody levels were detected in all the treated and recovered animals 74 (100%). The anti *T. parva* antibody responses generated were due to exposure to field parasites as none of the study animals had been vaccinated using ITM. Some of the sera from clinically healthy cattle showed sero-conversion to *T. parva* antigens indicating the prevalence and endemicity of ECF in the study area. The majority of study animals in all farms indicated an increase in the antibodies generated during the six-month follow-up period. The fact that all ECF clinically detected cattle were reported to have high antibody titers (PP ≥ 50%) at the end of the study suggests that treatment and recovery was an effective method of protecting cattle by sero-conversion. Similarly, some study animals that were also not clinically detected were sero-positive at the end of the study indicating a carrier status and disease endemicity in the study area. Similar studies in Tanzania showed the increased antibody levels in the treated and recovered Zebu calves [13]. 

Correlation between occurrence of specific antibodies to *T. parva* and degree of resistance to ECF have been previously described [19]. Similarly, other studies on the immunology of *T. parva* infections and the application of molecular tools led to the conclusion that sera of recovered animals neutralize sporozoites of various isolates [20]. The ELISA has been estimated to have a sensitivity of 99% and a specificity of 94% and 98% [11]. It is also known that cattle that recover from ECF either naturally or after treatment acquire resistance against subsequent similar parasite challenges. The protection that is induced is life-long, provided a constant parasite challenge occurs through repeated tick feeding. However, the acquired protection is not passed on to the calves in the colostrum. Thus, calves borne by immune cows are fully susceptible to ECF and this was the target age group for this study and clinical ECF was observed in the calves. Treatment by anti-theilerial drugs is therefore essential in the management and control of ECF in endemic areas in young calves that co-graze with the dams post-weaning. The absence of disease recurrences and ECF mortalities in calves during the follow-up period in the present study is clearly indicative of treatment efficacy and protective immunity. We, therefore, confirm the value of ECF clinically made diagnosis and recommend that early diagnosis and early treatment should be considered as an integral approach for the routine management of ECF since it is feasible and can serve as an alternative method to the currently used ITM for immunization against ECF in endemic areas. 

The early diagnosis and early treatment will be particularly effective for extensively managed herds where ITM has not been routinely used. The early diagnosis and early treatment approach should focus on young calves that require a small quantity (dose) of curative drugs and the costs attributable to delivery of the ITM are not incurred. The approach will also offer the benefits of use of local *T. parva* strains by the natural tick infections, thereby eliminating the risk of introducing new strains from a live vaccine. The approach does not require cold chain facilities, unlike the ITM and thus better potential for adaptability in resource poor cattle farming communities with local cattle breeds in ECF endemic areas. The findings indicate that early diagnosis and early treatment should be integrated in the management of ECF for prompt recovery. The suggested alternative decision to ITM was natural infection and anti-theilerial treatment as a better economic option where vaccine costs are increased or with enhanced efficacy of the treatment to a level where no deaths occur [21]. This is contrary to our findings where the treatment efficacy of 100% was achieved in early natural infection and treatment intervention. Additionally, the treatment costs were low (estimated at $8 per calf) based on lower dosage which corresponds with the small animal body size. 

Generally, none of the available control methods on their own will result in effective control of ECF. An integrated approach will always be required. If the disease becomes contained in given areas, eradication can be considered. However, this will be extremely difficult for areas where the vector tick (*R. appendiculatus*) is well established and where surveillance and diagnosis are inadequate. There is therefore an urgent need for an integrated control approach using the various prevailing methods. The core of early diagnosis and early treatment of ECF, which was assessed in this study, is to allow infected and recovered animals to develop protective immunity. This study simulated the only available ITM of ECF vaccination. Cattle were infected by sporozoites naturally under field conditions by the ticks, then early ECF disease diagnosis was done based on clinical symptoms and controlled by early treatment using the curative drug, Buparvaquone (Butalex^R^). The animals that recovered were protected from the subsequent homologous challenges and the immunity was continuously boosted by the vector ticks as they were naturally feeding on the cattle. 

However, negative control animals were not used in this study of early diagnosis and disease treatment of *T. parva* since the animals belonged to the farmers who had an economic value attachment to their animals. The negative control group can be used in future studies ensuring the study animals strictly belong to the research team and are not owned by the farmers. 

## 5. Conclusions

Early diagnosis and early treatment should be integrated in the routine management of ECF for prompt recovery with Buparvaquine in local cattle breeds in endemic areas. This will help farmers to reduce on calf mortality and acaricide application frequency in the indigenous cattle if it is integrated in the routine T&TBDs control program. 

## Figures and Tables

**Table 1:** Farmer socio-demographic data and farm characteristics.

Demographic Characteristics	Category	Frequency (%)
**Age (years)**	<40	17.6
	40–59	58.8
	>59	23.5
**Marital status**	Married	88.9
	Not-married	11.1
**Education level**	Non-formal	11.1
	Primary	38.9
	Secondary	11.1
	Tertiary	38.9
**Occupation**	Employed	5.6
	Livestock	94.4
**Cattle keeping period (years)**	5–10	11.1
	10–20	50.0
	>20	38.9
**Cattle system management**	Semi-intensive	17.9
	Extensive	82.4

**Table 2:** Response to ECF treatment in relation to clinical category before treatment.

ECF Clinical Category	Response to Anti-Theilerial Treatment		Total (%)
	Prompt/Rapid	Slow/Sluggish	
**Mild**	18 (100%)	0 (0%)	18 (24.3%)
**Moderate**	52 (92.9%)	4 (7.1%)	56 (75.7%)
**Total (%)**	70 (94.6%)	4 (5.4%)	74 (100)

**Table 3:** East Coast Fever prevalence and comparative analysis of microscopy, PCR and ELISA results at baseline.

Number of Positive Samples	Microscopy	PCR	ELISA
**ECF prevalence**	9 (3.7%)	76 (31.1%)	93 (38.1%)
**Concordant**			
**9 samples**	Positive	Positive	Positive
**Discordant**			
**67 samples**	Negative	Positive	Positive
**17 samples**	Negative	Negative	Positive

NB: All the positive samples by microscopy were also positive by PCR and ELISA. Similarly, all positive samples by PCR were positive by ELISA.

## Data Availability

The datasets used to analyse during current study are available from the corresponding author on request.
